# Identification of proteasome and caspase inhibitors targeting SARS-CoV-2 M^pro^

**DOI:** 10.1038/s41392-021-00639-8

**Published:** 2021-06-01

**Authors:** Zhengyuan Wang, Yao Zhao, Qingxing Wang, Yangfei Xing, Lu Feng, Juan Kong, Chao Peng, Leike Zhang, Haitao Yang, Min Lu

**Affiliations:** 1 Shanghai Institute of Hematology, State Key Laboratory of Medical Genomics, National Research Center for Translational Medicine (Shanghai), Ruijin Hospital affiliated to Shanghai Jiao Tong University School of Medicine, Shanghai, China; 2 Shanghai Institute for Advanced Immunochemical Studies and School of Life Science and Technology, ShanghaiTech University, Shanghai, China; 3 State Key Laboratory of Virology, Wuhan Institute of Virology, Center for Biosafety Mega-Science, Chinese Academy of Sciences, Wuhan, China; 4 National Facility for Protein Science in Shanghai, Zhangjiang Lab, Shanghai Advanced Research Institute, Chinese Academy of Science, Shanghai, China

**Keywords:** Structural biology, Drug screening

**Dear Editor,**

Since the beginning of 2020, the Coronavirus (CoV) Disease 2019 (COVID-19) pandemic has posed formidable challenges to public health security. The main protease (M^pro^, 3CL^pro^) of CoVs plays essential roles in viral replication, making them attractive targets for antiviral drug development^1–3^. Dozens of SARS-CoV-2 M^pro^ inhibitors have been reported with some entering clinical trials, but none is approved for COVID-19 treatment to date^1–3^. In this study, we discovered that the proteasome inhibitor MG132 and caspase inhibitors such as Z-VAD(OMe)-FMK are effective SARS-CoV-2 M^pro^ inhibitors.

We recently identified arsenic trioxide as an effective mutant p53 rescue compound functioning by increasing protein thermostability^4^. Here we used protein thermostability as a readout to screen SARS-CoV-2 M^pro^-thermostabilizing compounds from a library of 4198 chemical entities containing US Food and Drug Administration (FDA)-approved drugs and clinical-stage or known-target compounds (Supplementary Table S1). In the differential scanning fluorimetry (DSF) assay, Z-VAD(OMe)-FMK, MG132, boceprevir, thermopsine, and baicalein were identified as the top five SARS-CoV-2 M^pro^-thermostabilizing compounds, which increased the melting temperature (*T*_m_) of M^pro^ by at least 2 °C (Fig. 1a, Supplementary Fig. S1a, and Supplementary Table S1). The destabilizing hits (Fig. 1a and Supplementary Fig. S1b) were not pursued further because of their potential promiscuity of binding or other undesirable properties. The top five M^pro^-stabilizing compounds were validated in concentration titration, whereby Z-VAD(OMe)-FMK was consistently the most potent M^pro^-stabilizing compound (Supplementary Fig. S1c, d).

**Fig. 1:**
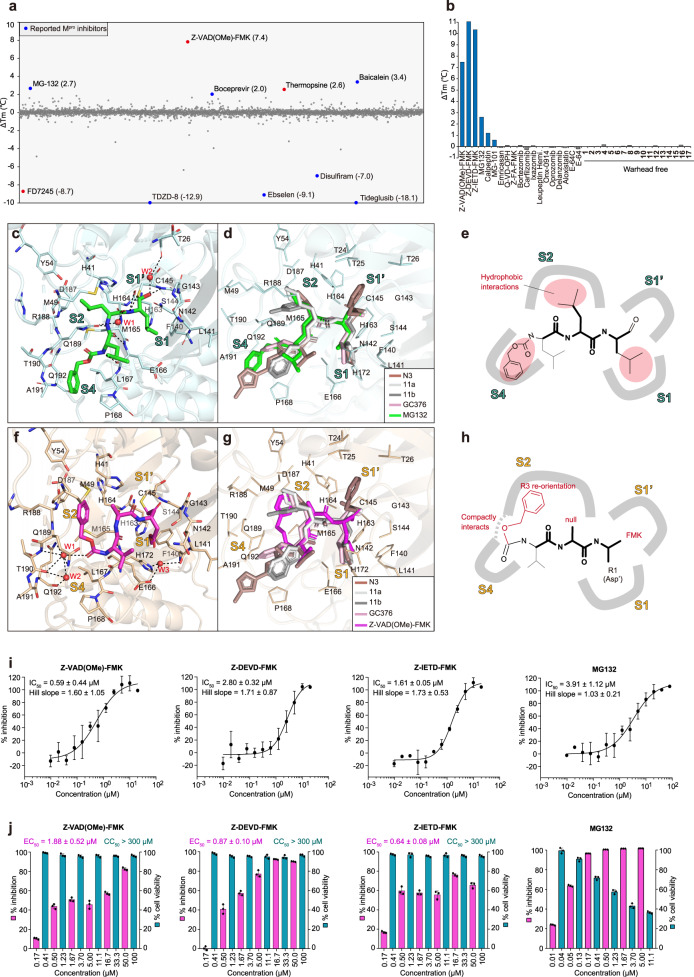
Identification of MG132 and Z-VAD(OMe)-FMK as inhibitors targeting SARS-CoV-2 M^pro^. **a** Dot profile showing the change of *T*_m_ (Δ*T*_m_) of SARS-CoV-2 M^pro^ upon incubation with 4198 compounds in the pilot DSF screening (*n* = 1). Values in brackets show the Δ*T*_m_ induced by the indicated compounds. The top five stabilizing and destabilizing hits are shown. **b** Bar graph showing the Δ*T*_m_ of M^pro^ induced by the 36 studied compounds (1:10 molar ratio of M^pro^:compound). **c** Close-up view of the binding mode of MG132 in the active site of SARS-CoV-2 M^pro^. The M^pro^ residues involved in MG132 binding are shown as pale cyan sticks. Hydrogen bonds are indicated with dashed lines. **d** Comparison of the binding mode of MG132 in the active site of SARS-CoV-2 M^pro^ with those of 11a (PDB code: 6LZE), 11b (PDB code: 6M0K), GC376 (PDB code: 6WTT), and N3 (PDB code: 6LU7). **e** Summarized binding mode of MG132 in the active site of SARS-CoV-2 M^pro^. The pink areas represent hydrophobic interaction networks between M^pro^ subsites and MG132. **f** Close-up view of the binding mode of Z-VAD(OMe)-FMK in the active site of SARS-CoV-2 M^pro^. The residues involved in Z-VAD(OMe)-FMK binding are shown as beige sticks. Hydrogen bonds are indicated with dashed lines. **g** Comparison of the binding mode of Z-VAD(OMe)-FMK in the active site of SARS-CoV-2 M^pro^ with those of 11a (PDB code: 6LZE), 11b (PDB code: 6M0K), GC376 (PDB code: 6WTT), and N3 (PDB code: 6LU7). **h** Summarized binding mode of M^pro^-Z-VAD(OMe)-FMK. The red labels represent the unique binding features. **i** The hydrolytic activity of SARS-CoV-2 M^pro^ was measured in the presence of increasing concentrations of the indicated compounds. The IC_50_ values were determined based on dose–response curves using nonlinear regression. The data represent mean ± SEM from 3 experiments. The *y*-axis of the graphs represents mean % inhibition of hydrolytic activity. **j** Antiviral activity against SARS-CoV-2 and cytotoxicity of the indicated compounds in Vero cells. Vero cells were treated with a concentration series of the indicated compounds and infected with SARS-CoV-2 (MOI = 0.01). At 24 h post-infection, the virions in the culture supernatant were harvested, followed by determination of the viral RNA copy number by qRT-PCR. The cytotoxicity of the indicated compounds in Vero cells was determined using the CCK8 assay. The *y*-axes of the graphs represent mean % reduction of the virion yield or mean % cell viability.

We next predicted the M^pro^-binding modes of the five hits and performed a structure–activity relationship (SAR) analysis. The substrate-binding pocket of M^pro^, containing four subsites (S1′, S1, S2, and S4), is highly conserved among all CoV M^pro^ homologs (Supplementary Fig. S2a, upper panel). We previously designed a series of M^pro^ inhibitors harboring a classic core structure, in which the four subgroups (warhead, R1, R2, and R3) were assigned to occupy the four subsites of M^pro^, respectively (Supplementary Fig. S2a, lower panel)^2^, as exemplified by N3^1^, 11a^2^, GC376^3^, and MI-23^5^ (Supplementary Fig. S2b). Among the top five hits, baicalein and thermopsine are natural products with low molecular weight (Supplementary Fig. S1d), and thus they are unlikely to compactly occupy all four subsites. Boceprevir has been reported to function based on the classic core structure^3^, and its M^pro^-binding mechanism has been elucidated^5^. The remaining two hits, MG132 and Z-VAD(OMe)-FMK, structurally harbor a classic core structure and were thus proposed to bind to the active site of M^pro^ in a conventional binding mode (Supplementary Fig. S2c). Notably, Z-VAD(OMe)-FMK lacks an R2 subgroup in the proposed binding mode (Supplementary Fig. S2c). To test the proposed binding mode, we collected 34 commercially available compounds sharing high structural similarity with MG132 and Z-VAD(OMe)-FMK (Supplementary Fig. S2d). Among them, three caspase inhibitors and three proteasome inhibitors detectably stabilized M^pro^ (Fig. 1b, the 6 blue bars, Δ*T*_m_ > 0.5 °C). The 17 compounds (**1**–**17**) lacking cysteine-binding warhead (Supplementary Fig. S2d) failed to stabilize M^pro^ (Fig. 1b, Δ*T*_m_ < 0.1 °C). We next focused on caspase and proteasome inhibitors and collected 25 commercially available inhibitors (Supplementary Fig. S2e). The DSF results suggested: (1) harboring core structure is apparently a prerequisite for being a competent M^pro^ thermostabilizer (the 8 blue bars); (2) the top 5 potent compounds all contain a fluoromethyl ketone (FMK) warhead (the first 5 bars); and (3) small-sized R2 is apparently associated with high potency (comparison among the first 6 bars). In summary, the three most potent Z-VAD(OMe)-FMK, Z-DEVD-FMK, and Z-IETD-FMK contain a core structure with an FMK warhead and a small-sized R2.

To validate that M^pro^ is a direct target of MG132 on the atomic level, we solved a high-resolution crystal structure of M^pro^-MG132 (Supplementary Table S2; 1.7 Å resolution). The two M^pro^ molecules formed a symmetry homodimer (Supplementary Fig. S3a). As proposed in Fig. S2c, MG132 binds to the active site based on the classic core structure (Supplementary Fig. S3b), whereby an aldehyde, side chain of Leu, side chain of the second Leu, and benzyloxycarbonyl (Cbz) act as the warhead, R1, R2, and R3, respectively (Fig. 1c). Mass spectrometry (MS) did not reveal an obvious molecular weight increase of M^pro^ upon incubation with MG132 (Supplementary Fig. S3c; ebselen was used as a control), which is presumably due to the highly dynamic and reversible bond between the MG132 aldehyde and the targeted cysteine. The mode of MG132 binding to SARS-CoV-2 M^pro^ significantly differs from that of its binding to the proteasomal 20S subunit because of the different shape of the binding pocket (Supplementary Fig. S3d). Nevertheless, it is similar to those of the reported classical core-structure-based M^pro^ inhibitors (Fig. 1d). Notably, MG132 subgroups R1, R2, and R3 are all derived from hydrophobic Leu and Cbz, which undergo extensive hydrophobic interactions with M^pro^ (Fig. 1e). The core structure of MG132 forms four hydrogen bonds with M^pro^ (comparable with the reported inhibitors), whereby the R1, R2, and R3 subgroups form only one hydrogen bond with M^pro^ (Supplementary Fig. S3e).

We next solved a high-resolution crystal structure of the M^pro^-Z-VAD(OMe)-FMK complex (Supplementary Table S2; 1.8 Å resolution), wherein the asymmetric unit contains one molecule (Supplementary Fig. S4a). Z-VAD(OMe)-FMK binds to the active site of M^pro^ (Supplementary Fig. S4b), however, in an unexpected binding mode (Fig. 1f). The FMK group acts as a previously unreported Cys145-binding warhead, which was confirmed by MS (Supplementary Fig. S4c, d; Z-DEVD-FMK and Z-IETD-FMK with the FMK group were also confirmed to covalently bind to Cys145). FMK is frequently used as a warhead to tether cysteines of caspases. However, it has not been reported to be used in SARS-CoV-2 M^pro^ inhibitors, to our knowledge. The binding mode of M^pro^-Z-VAD(OMe)-FMK differs significantly from that of caspase-1-Z-VAD-FMK, whereby the Z-VAD(OMe)-FMK molecule linearly occupies the long narrow pocket of caspase-1 (Supplementary Fig. S4e). Due to R3 re-orientation, the binding mode of Z-VAD(OMe)-FMK to M^pro^ is completely different from that of previously reported M^pro^ inhibitors (Fig. 1g). The four features of M^pro^-Z-VAD(OMe)-FMK binding (Fig. 1h)—FMK acting as the warhead, absence of R2, re-orientation of flexible hydrophobic R3, and compact occupation of the S2-S4 joint site—may contribute to the high potency of Z-VAD(OMe)-FMK in thermostabilizing SARS-CoV-2 M^pro^. During preparation of this manuscript, a crystal structure of M^pro^-Z-VAD(OMe)-FMK (PDB ID: 7C8B) was released in the PDB database by an independent group, confirming the observed unconventional binding mode.

The inhibitory activities of the three caspase inhibitors and MG132 against SARS-CoV-2 M^pro^ were determined in vitro using a fluorescence resonance energy transfer (FRET)-based assay. The three caspase inhibitors exhibited potent M^pro^ inhibitory effects with half-maximal inhibitory concentrations (IC_50_) in the nanomolar and low micromolar range (Fig. 1i, 0.59–2.80 μM), while MG132 displayed a higher IC_50_ (3.91 μM). Generally speaking, the three caspase inhibitors were comparable in their IC_50_ values with the reported rationally designed M^pro^ inhibitors (Supplementary Fig. S5a, 0.03–30.0 μM).

The antiviral activities of these four compounds against SARS-CoV-2 were next determined in Vero cells. At 24 h following SARS-CoV-2 infection, the three caspase inhibitors displayed potent half-maximal effective concentrations (EC_50_) in the nanomolar and low micromolar range in Vero cells (Fig. 1j, 0.64–1.88 μM). We noticed that Z-VAD(OMe)-FMK exhibited higher potency in enzymatic activity assay, whereas lower potency in antiviral activity assay when compared to the other two caspase inhibitors. It may be associated with potential ‘off-target’ effect of Z-VAD(OMe)-FMK when inhibiting M^pro^ in the cell-based antiviral assay, for example, promiscuously binding to multiple caspases and proteins of host cells. None of the tested caspase inhibitors caused cytotoxicity in Vero cells, exhibiting half-maximal cytotoxic concentrations (CC_50_) > 300 μM (Fig. 1j). MG132 is cytotoxic to this cell line^3^, and its EC_50_ could not be reliably determined (Fig. 1j). The extended cytotoxicity studies consistently suggested that these three caspase inhibitors were relatively non-toxic to cells (Supplementary Fig. S5b). Compared to the anti-SARS-CoV-2 activities of the reported M^pro^ inhibitors measured in the same Vero cells, the non-toxic caspase inhibitors were superior to those of N3^1^, Boceprevir^3^, and GC-376^3^, comparable with the six most potent M^pro^ inhibitors (MI-09/12/14/28/30/31) reported by Qiao and colleagues^5^, but less potent than our previously optimized 11a/b^2^ (Supplementary Fig. S5a).

In summary, we identified MG132, Z-VAD(OMe)-FMK and its structural analogs as direct M^pro^-binding small molecules with antiviral activity against SARS-CoV-2. MG132 is widely recognized as a CoV inhibitor, with several reported and proposed targets. Our study provides evidence at single-atom resolution that M^pro^ is a direct target of MG132. In contrast to the reported rationally designed M^pro^ inhibitors, Z-VAD(OMe)-FMK binds to the active site of M^pro^ in an unconventional binding mode, which may contribute to the observed high potency of Z-VAD(OMe)-FMK in inhibiting SARS-CoV-2. Our studies provide compelling structural evidence to support an alternative strategy for the design of potent inhibitors against SARS-CoV-2 M^pro^.

## Supplementary information

Supplementary material.

Table S1.

Table S2.

## Data Availability

The datasets generated in this study are available from the corresponding authors upon reasonable request.

## Code availability

Atomic coordinates and structure factors of the reported crystal structures have been deposited with the worldwide Protein Data Bank (https://www.rcsb.org) under the PDB codes 7CUT and 7CUU.
