# Supplementary material for: Identification of proteasome and caspase inhibitors targeting SARS-CoV-2 Mpro
Source: Signal Transduct Target Ther. 2021 Jun 1;6:214. doi: 10.1038/s41392-021-00639-8 (PMC8166895; doi:10.1038/s41392-021-00639-8)
Supplement: Supplementary file 1 — Supplementary material. [file 41392_2021_639_MOESM1_ESM.pdf]

Supplementary Materials for

Identification of proteasome and caspase inhibitors targeting SARS-CoV-2 M^{pro}

Zhengyuan Wang[1*], Yao Zhao[2*], Qingxing Wang[3*], Yangfei Xing[1*], Lu Feng[2], Juan Kong[2], Chao Peng[4], Leike Zhang[3#], Haitao Yang[2#], Min Lu[1#]

[1]Shanghai Institute of Hematology, State Key Laboratory of Medical Genomics, National Research Center for Translational Medicine (Shanghai), Ruijin Hospital affiliated to Shanghai Jiao Tong University School of Medicine, Shanghai, China.

[2]Shanghai Institute for Advanced Immunochemical Studies and School of Life Science and Technology, ShanghaiTech University, Shanghai, China.

[3]State Key Laboratory of Virology, Wuhan Institute of Virology, Center for Biosafety Mega-Science, Chinese Academy of Sciences, Wuhan, China.

[4]National Facility for Protein Science in Shanghai, Zhangjiang Lab, Shanghai Advanced Research Institute, Chinese Academy of Science, Shanghai, China.

* These authors contributed equally to this work.

#Correspondence to: Dr. Min Lu (min.lu@shsmu.edu.cn) or Dr. Haitao Yang (yanght@shanghaitech.edu.cn) or Dr. Leike Zhang (zhangleike@wh.iov.cn)

This PDF file includes:

Materials and Methods
Fig. S1 to S5
Captions for Tables S1 to S2

Materials and Methods

Protein cloning, expression, and purification

Full length SARS-CoV-2 3C-like protease ($3CL^{pro}$) was cloned into pGEX-6p-1 vector, with a C-terminal His-tag. A linker (AVLQ) between GST and M^{pro} was designed to produce an autocleavage site for M^{pro}. The protein was expressed in E. coli BL21(DE3)-Gold cells (Stratagene). A single colony was inoculated in 100 mL LB media containing 100 ug/ml Ampicillin and shaken at 37 °C, 180 rpm overnight to produce the seeds. 20 mL seeds culture was added to 2L LB media containing 100 ug/ml Ampicillin and shaken at 37 °C, 180 rpm until OD600 reached ~0.4. The temperature was then lowered to 16 °C, and isopropyl 1-thio-β-D-galactopyranoside was added to a final concentration of 0.1 mM when OD600 reached ~0.6. Expression was continued at 16 °C, 180 rpm overnight. Cells were collected by centrifugation, and the pellet was resuspended in lysis buffer (25 mM Tris, 300 mM NaCl, 1 mM PMSF, pH 8.0) and sonicated. Supernatant was separated from cell debris by centrifugation at 10,000 g for 30 min at 4 °C and loaded onto a Ni-NTA column (Qiagen) that equilibrated with lysis buffer. The column was washed by lysis buffer with additional 20 mM imidazole and eluted by lysis buffer with 250 mM imidazole. The target protein from elute fraction was digested by r3C protease at a ratio of 1:100 and dialyzed against lysis buffer at 4 °C overnight. The tag-removed M^{pro} was loaded onto a 2nd Ni-NTA column (Qiagen) that equilibrated with lysis buffer. The column was washed by lysis buffer with 20 mM imidazole and eluted by lysis buffer with 250 mM imidazole. The protein from flowthrough fraction was concentrated and loaded onto a Superdex 75 column (GE Healthcare) pre-equilibrated with storage buffer (50 mM Tris, 1 mM EDTA, pH 7.3). The fractions with high purity were pooled and concentrated to a final concentration of 10 mg/ml. The protein was flash-frozen by liquid N2 and stored at -80°C.

Protein crystallization, data collection and structure determination

5 mg/ml M^{pro} in storage buffer was incubated with 1 mM compounds on ice for 2 hr before setting up broad crystal screen. The screen was set up with sitting-drop vapor diffusion method by mixing 150 nl protein with 150 nl well solution. For M^{pro}-Z-VAD(OMe)-FMK, the co-crystal was obtained after 2 days at 4°C and from the well solution 0.1 M HEPES pH 7.5, 10% w/v PEG6000, 5% v/v MPD. For M^{pro}-MG132, the co-crystal was obtained after 9 days at 18°C and from the well solution 0.1 M HEPES pH 7.5, 10% w/v PEG4000, 5% v/v Isopropanol. For M^{pro}-Z-VAD(OMe)-FMK and M^{pro}-MG132, X-ray data were collected on beamline BL17U1 and BL19U1 at Shanghai Synchrotron Radiation Facility (SSRF) at 100 K and with Eiger X 16M image plate detector and Pilatus3 6M image plate detector. Data integration and scaling were performed using the program XDS. Both structures were determined by molecular replacement (MR) with the PHASER and Phenix 1.18.2 using the COVID-19 virus M^{pro} (PDB ID: 6LU7) as a search template. Models from MR were subsequently subjected to iterative cycles of manual model adjustment with Coot 0.8 and refinement was completed with Phenix REFINE. The inhibitors Z-VAD(OMe)-FMK and MG132 were built according to the omit map. The phasing and refinement statistics are summarized in Table S2. Coordinates and structure factors have been deposited in Protein Data Bank (PDB) with accession number 7CUT and 7CUU.

DSF-based library screening

Potential inhibitors thermostabilizing SARS-CoV-2 M^{pro} recombinant protein were screened by DSF. Two drug libraries - Bioactive Compound Library (Target Mol, #L4000) and FDA-approved Drug Library (Selleck, #L2000)-that together comprised 4198 compounds were applied in screen.

In a single well of a 384-well PCR plate, a 10 μL reaction was conducted by combing 8 μL of protein solution in Analysis Buffer (20 mM HEPES pH 7.5), 2 μL of 25 X SYPRO® orange (diluted from 5000 X stock with Analysis Buffer) and 0.1 μL of solution containing either 10 mM compound or DMSO (as vehicle controls). The final conditions in the experimental well were 5 X SYPRO® orange, 10 μM compound (if any), 1 μM purified recombinant SARS-CoV-2 M^{pro} and 2% (v/v) DMSO in Analysis Buffer. DSF signal was measured using LightCycler® 480 RT-PCR machine by increasing the temperature of plate by 2 °C per minute from 30-90 °C, taking a fluorescence reading every 0.2 °C using a LED/photodiode set matched to the excitation and emission wavelengths of SYPRO® orange. *Tm* values were analyzed using Roche LightCycler® Thermal Shift Analysis software by fitting the raw fluorescence data to a Boltzmann sigmoidal curve. Δ*Tm* values were calculated by subtracting the average *Tm* of the all samples in a same plate.

Intact protein analysis

2.5 μL of compounds (10 mM in DMSO) were add into 50uL of the proteins (3 mg/mL). The mixtures were kept in room temperature for 30 min. Liquid chromatography−mass spectrometry (LC−MS) analyses were performed in positive-ion mode with an Agilent 6550 quadrupole-time-of-flight (QTOF) mass spectrometer (Santa Clara, CA) coupled with an Agilent 1260 high-performance liquid chromatograph (HPLC; Santa Clara, CA) for detecting the molecular weight of intact proteins. The samples were eluted from a Phenomenex Jupiter C4 300Å LC Column (2×150 mm, 5μm) over a 15 min gradient from 5% to 100% acetonitrile containing 0.1% formic acid at a flow rate of 0.5 mL/min. The acquisition method in positive-ion mode with Dual Agilent Jet Stream electrospray voltage used a capillary temperature of 250 °C, a fragmentor of 175 V, a capillary voltage of 3000 V. Mass deconvolution was performed using Agilent MassHunter Qualitative Analysis B.06.00 software with BioConfirm Workflow.

Tandem MS/MS analysis

The samples were precipitated and resolved by 8 M urea, and then digested for 16 h at 25 °C by chymotrypsin at an enzyme-to-substrate ratio of 1:50 (wt/wt). The digested peptides were desalted and loaded onto a homemade 30 cm-long pulled-tip analytical column (ReproSil-Pur C18 AQ 1.9μm particle size, Dr. Maisch GmbH, 75 μm ID× 360 μm OD) connected to an Easy-nLC1200 UHPLC (Thermo Scientific) for MS analysis. The elution gradient and mobile phase constitution used for peptide separation were as follows: 0-1 min, 4%-8% B; 1-96 min, 8-35% B; 96-104 min, 35-60% B; 105-120min, 60-100% B (mobile phase A: 0.1% Formic Acid in Water and mobile phase B: 0.1% formic acid in 80% Acetonitrile) at a flow rate of 300 nL/min. Peptides eluted from the LC column were directly electro-sprayed into the mass spectrometer with the application of a distal 1.8-kV spray voltage. Survey full-scan MS spectra (from m/z 300–1800) were acquired in the Orbitrap analyzer (Q Exactive) with resolution r =70,000 at m/z 400. And top 20 MS/MS events were sequentially generated selected from the full MS spectrum at a 30% normalized collision energy. The dynamic exclusion time was set at 10 seconds. One acquisition cycle includes one full-scan MS spectrum followed by top 20 MS/MS events, sequentially generated on the first to the twentieth most intense ions selected from the full MS spectrum at a 28% normalized collision energy. The acquired MS/MS data were analyzed using UniProtKB E.coli database (database released on Nov. 11, 2016) containing M^{pro} using Protein Discoverer 2.1. In order to accurately estimate peptide probabilities and false discovery rates (FDR), we used a decoy database containing the reversed sequences of all the proteins appended to the target database. FDR was set

at 0.01. Mass tolerance for precursor ions was set at 20ppm. Chymotrypsin was defined as cleavage enzyme and the maximal number of missed cleavage sites was set at 4. Protein N-terminus acetylation, methionine oxidation and compounds covalent bindings were set as variable modifications. The modified peptides were manually checked and labeled.

Enzymatic activity and inhibition assays

Enzyme activity inhibition assays were mainly based on the previously report[1-3]. Briefly, IC_{50} values of the tested compounds were calculated from enzyme activity inhibition assays using 0.2 μM SARS-CoV-2 M^{pro} protein, 20 μM substrate and at least 11 different compound concentrations. Data was derived from three independent experiments and analyzed using GraphPad Prism (log(inhibitor) vs. response -- Variable slope).

Antiviral and cytotoxicity assays

Antiviral and cytotoxicity assays were mainly based on the previously report[1-3]. Briefly, Vero cells (1×10^5 cells) were treated with different doses of compound and viruses (MOI = 0.01) for 24 hrs. Culture supernatants were harvested and SARS-CoV-2 vRNA was extracted using TaKaRa MiniBEST Viral RNA/DNA Extraction Kit Ver.5.0 (TaKaRa, #9766) following manufacturer's instructions. vRNA was reverse transcribed into cDNA using PrimeScript™ RT reagent Kit with gDNA Eraser (TaKaRa, # RR047A). 1 μL of cDNA was used for real-time PCR assay using TB Green® Premix Ex Taq™ II (Tli RNaseH Plus) (TaKaRa, #RR820A). vRNA copies per μL were determined using plasmid containing the spike protein gene of SARS-CoV-2. Primers for quantifying SARS-CoV-2 are 5′-CAATGGTTTAACAGGCACAGG-3′ and 5′-CTCAAGTGTCTGTGGATCACG-3′. The cytotoxicity of compounds on all cell lines was determined by CCK-8 assays (Beyotime, #C0039). The infection experiments were performed at BSL-3. All experiments were performed in triplicates. EC_{50} values of the test compounds were calculated from antiviral assays using the nonlinear regression model in GraphPad Prism (log(agonist) vs. response -- Variable slope (four parameters)).

References:

1 Zhang, L. *et al.* Crystal structure of SARS-CoV-2 main protease provides a basis for design of improved α-ketoamide inhibitors. *Science (New York, N.Y.)* **368**, 409-412, doi:10.1126/science.abb3405 (2020).

2 Dai, W. *et al.* Structure-based design of antiviral drug candidates targeting the SARS-CoV-2 main protease. *Science (New York, N.Y.)* **368**, 1331-1335, doi:10.1126/science.abb4489 (2020).

3 Jin, Z. *et al.* Structure of M(pro) from SARS-CoV-2 and discovery of its inhibitors. *Nature* **582**, 289-293, doi:10.1038/s41586-020-2223-y (2020).

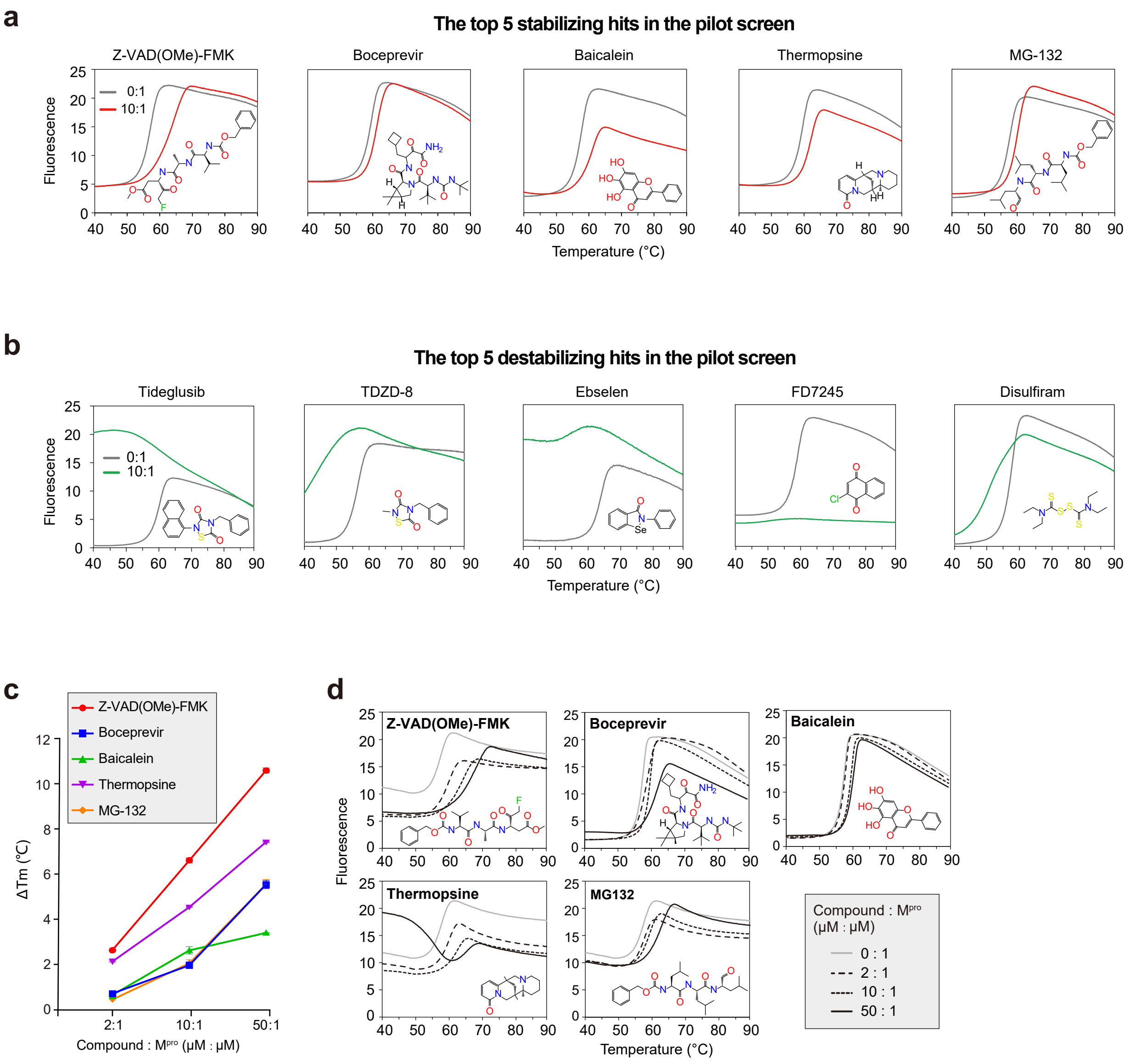
a
The top 5 stabilizing hits in the pilot screen
Z-VAD(OMe)-FMK
Boceprevir
Baicalein
Thermopsine
MG-132
0:1
10:1
Fluorescence
Temperature (°C)
b
The top 5 destabilizing hits in the pilot screen
Tideglusib
TDZD-8
Ebselen
FD7245
Disulfiram
0:1
10:1
Fluorescence
Temperature (°C)
c
Z-VAD(OMe)-FMK
Boceprevir
Baicalein
Thermopsine
MG-132
ΔTm (°C)
2:1
10:1
50:1
Compound : Mpro (μM : μM)
d
Z-VAD(OMe)-FMK
Boceprevir
Baicalein
Thermopsine
MG132
Fluorescence
Temperature (°C)
Compound : Mpro (μM : μM)
0 : 1
2 : 1
10 : 1
50 : 1

Fig. S1

Fig. S1

Screening for M^{pro}-stabilizing compounds. **a** The melting curves of M^{pro} upon incubation with the indicated top five stabilizing hits in the pilot screen as shown in Figure 1a. **b** The melting curves of M^{pro} upon incubation with the indicated top five destabilizing hits in the pilot screen as shown in Figure 1a. **c** Validation of the top five stabilizing hits. SARS-CoV-2 M^{pro} was mixed with the indicated compounds at the indicated ratios, followed by DSF determination. Graph shows the calculated ΔT_m (mean ± SD; n = 3). **d** Representative melting curves of M^{pro} upon incubation with the indicated compounds.

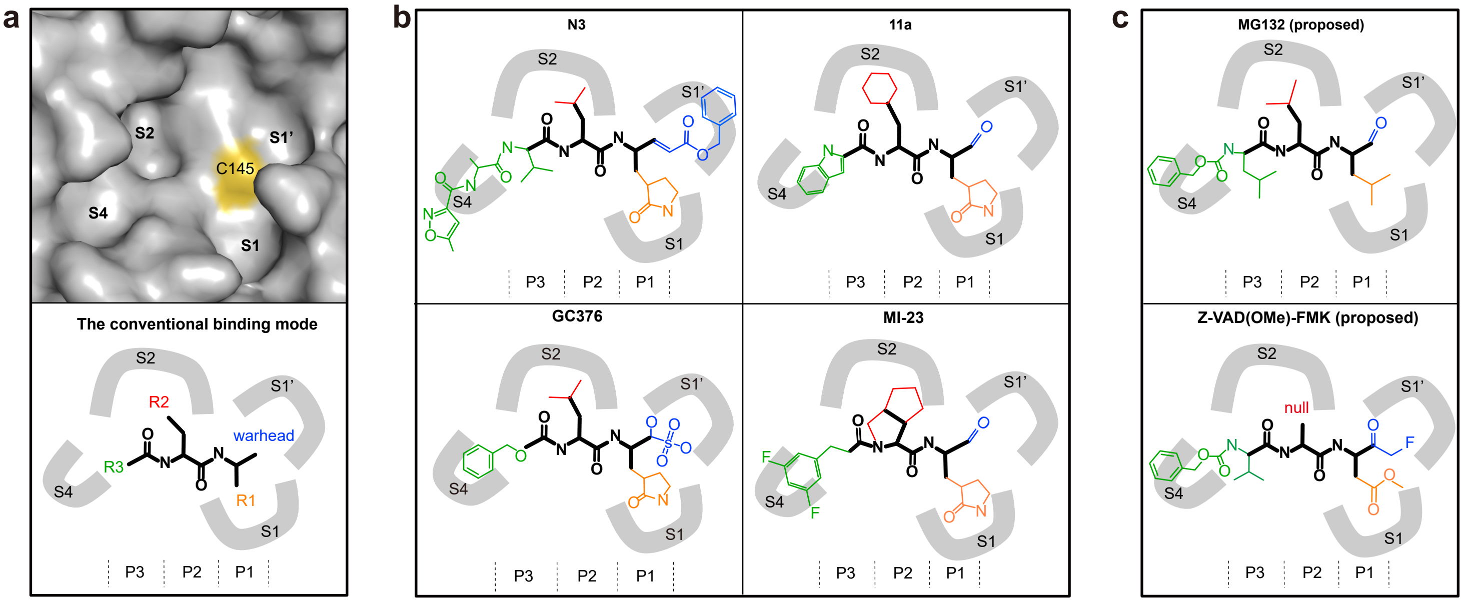

d

The 34 compounds sharing structural similarity with Z-VAD(OMe)-FMK and MG132

Name (CID #)	Warhead	R1	R2	R3	ΔTm
Z-VAD(OMe)-FMK (5497174)			null		7.4
Z-DEVD-FMK (16760394)			null *		11.1
Z-IETD-FMK (92043243)			null *		10.7
MG132 (462382)					2.7
Calpeptin (73364)					1.1
MG-101 (443118)					0.6
Emricasan (216325)			null		< 0.1
Q-VD-OPH (52938427)					< 0.1
Z-FA-FMK (6915837)		CH3			< 0.1
Bortezomib (387447)					< 0.1
Carfilzomib (11556711)					< 0.1
Ixazomib (25183872)			null		< 0.1
Onx-0914 (23642227)					< 0.1
Oprozomib (25067547)					< 0.1
Delanzomib (24800541)					< 0.1
E-64C (123664)	null				< 0.1
Aloxistatin (65663)	null				< 0.1
E-64 (123985)	null				< 0.1
Leupeptin Hemisulfate (2733491)					< 0.1

* atypical R2 at core structure

Name (CID #)	Warhead position	R1	R2	R3	ΔTm
1 (7289349)		NH2	null		< 0.1
2 (97991417)					< 0.1
3 (7289334)	NH2	CH3			< 0.1
4 (46499339)	NH2				< 0.1
5 (3103043)	NH2				< 0.1
6 (16760394)	NH2	CH3			< 0.1
7 (92224632)	NH2				< 0.1
8 (99735293)	NH2				< 0.1
9 (5233249)	NH2				< 0.1
10 (3103057)	NH2				< 0.1
11 (16760394)	NH2				< 0.1
12 (3103078)			null		< 0.1
13 (3103022)					< 0.1
14 (92216326)					< 0.1
15 (40632936)	HO		null		< 0.1
16 (40631979)	NH2		null		< 0.1
17 (7289345)	NH2		null		< 0.1

e

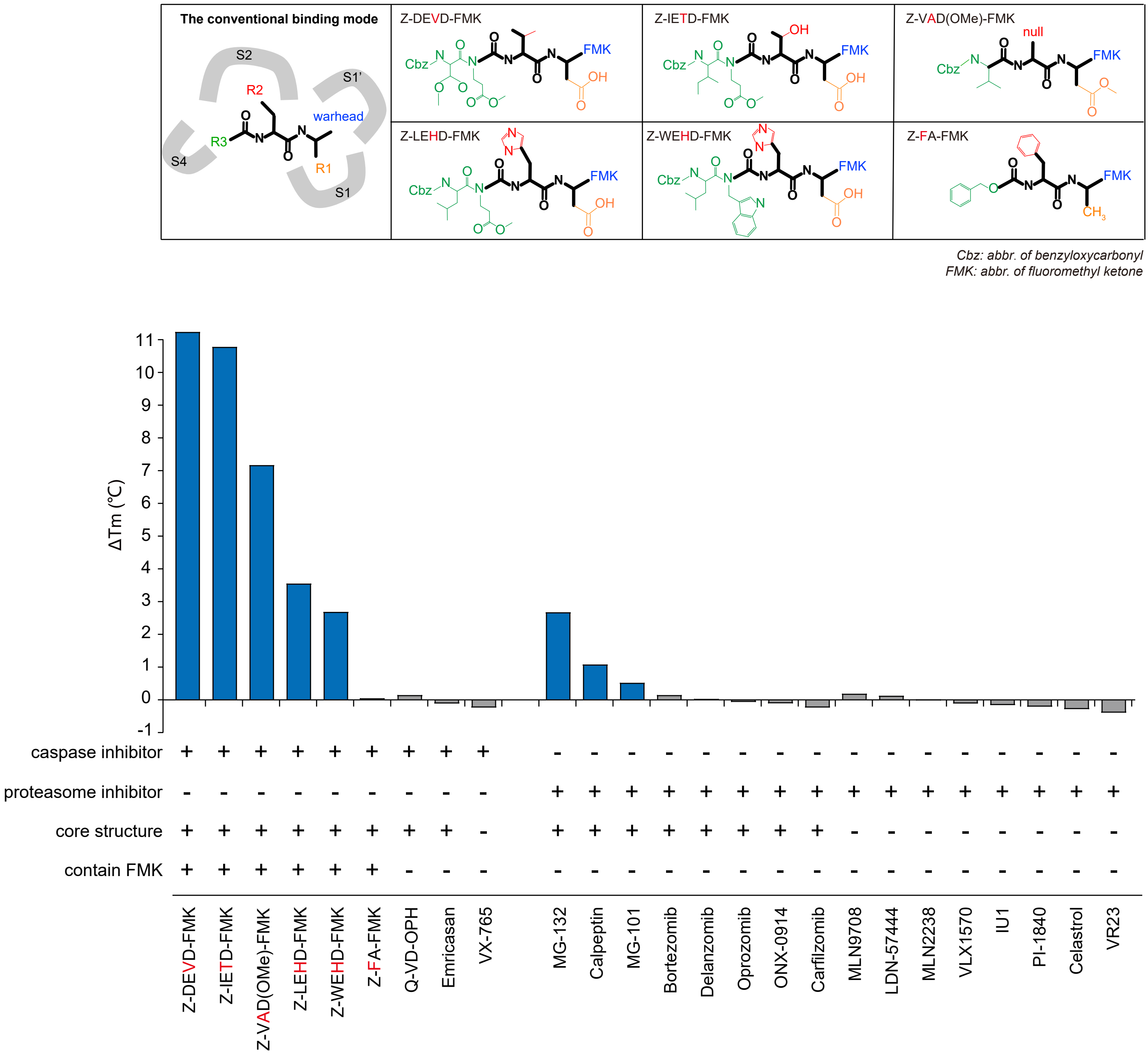

The conventional binding mode
S2
S1'
R2
warhead
R3
S4
R1
S1
Z-DEVD-FMK
Z-IETD-FMK
Z-VAD(OMe)-FMK
Z-LEHD-FMK
Z-WEHD-FMK
Z-FA-FMK
Cbz
FMK
OH
null
Cbz: abbr. of benzyloxycarbonyl
FMK: abbr. of fluoromethyl ketone
ΔTm (°C)
caspase inhibitor
proteasome inhibitor
core structure
contain FMK
Z-DEVD-FMK
Z-IETD-FMK
Z-VAD(OMe)-FMK
Z-LEHD-FMK
Z-WEHD-FMK
Z-FA-FMK
Q-VD-OPH
Emricasan
VX-765
MG-132
Calpeptin
MG-101
Bortezomib
Delanzomib
Oprozomib
ONX-0914
Carfilzomib
MLN9708
LDN-57444
MLN2238
VLX1570
IU1
PI-1840
Celastrol
VR23

Fig. S2

Fig. S2

Structure-activity relationship. **a** The active site of SARS-CoV-2 M^{pro} (upper panel) and the binding mode of the designed small molecules to this site (lower panel). **b** Four representatives of the reported SARS-CoV-2 M^{pro} inhibitors and their conventional M^{pro}-binding modes. **c** The proposed M^{pro}-binding modes of MG132 and Z-VAD(OMe)-FMK. **d** Table lists the subgroups of the 36 M^{pro}-stabilizing compounds tested in the structure-activity relationship. ΔTm induced are shown in the right column. **e** Bar graph showing ΔT_m of M^{pro} induced by the 25 indicated caspase and proteasome inhibitors (1:10 molar ratio of M^{pro}:compounds) and the structures of the 6 FMK-containing caspase inhibitors, wherein the R2 of the 6 FMK-containing compounds at P1 is labeled in red.

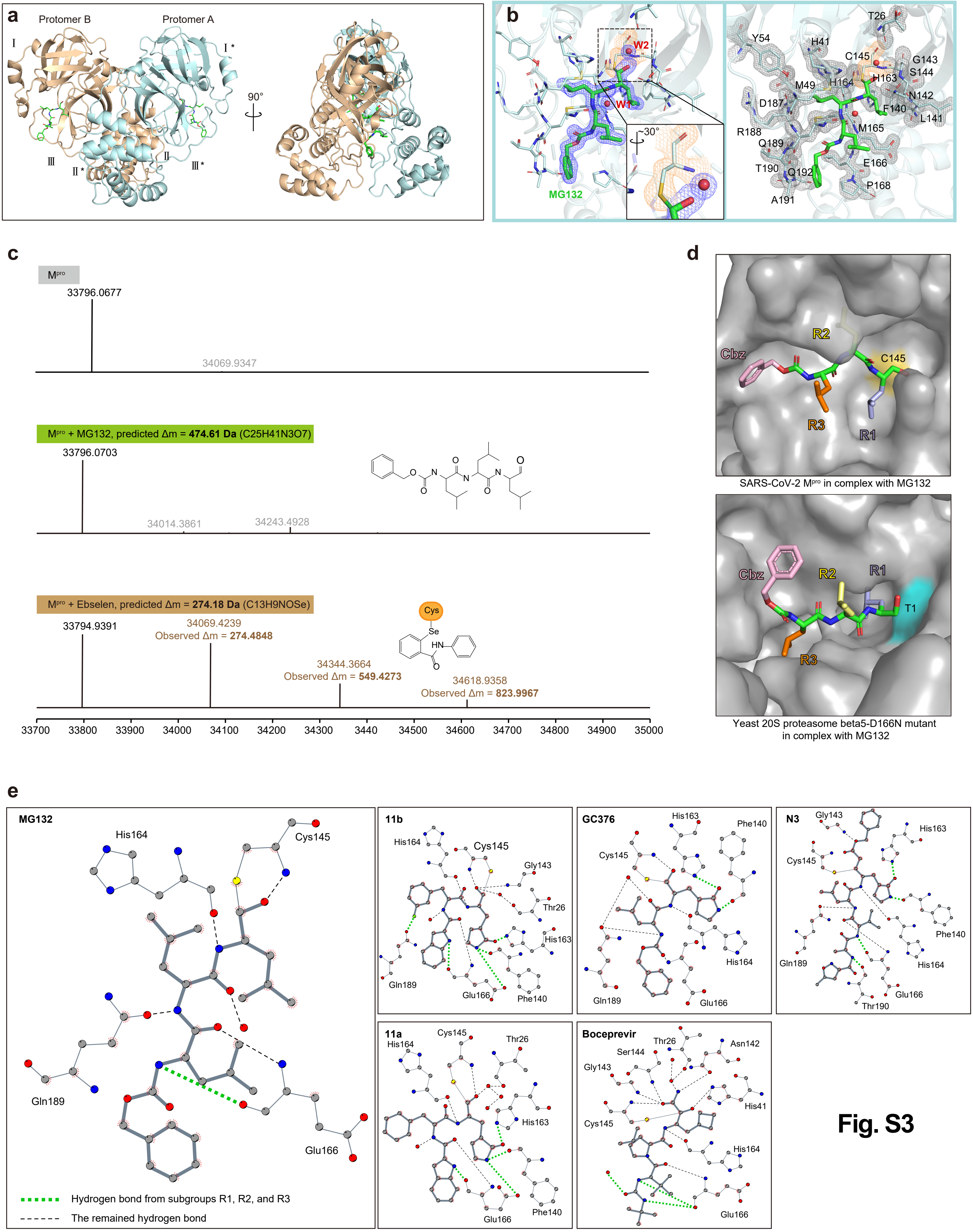
a
Protomer B
Protomer A
90°
b
W2
W1
MG132
~30°
T26
Y54
H41
C145
G143
S144
M49
H164
H163
N142
D187
F140
L141
R188
M165
Q189
E166
T190
Q192
P168
A191
c
Mpro
33796.0677
34069.9347
Mpro + MG132, predicted Δm = 474.61 Da (C25H41N3O7)
33796.0703
34014.3861
34243.4928
Mpro + Ebselen, predicted Δm = 274.18 Da (C13H9NOSe)
33794.9391
34069.4239
Observed Δm = 274.4848
34344.3664
Observed Δm = 549.4273
34618.9358
Observed Δm = 823.9967
Cys
Se
HN
33700 33800 33900 34000 34100 34200 34300 3440 34500 34600 34700 34800 34900 35000
d
Cbz
R2
C145
R3
R1
SARS-CoV-2 Mpro in complex with MG132
Cbz
R2
R1
T1
R3
Yeast 20S proteasome beta5-D166N mutant in complex with MG132
e
MG132
His164
Cys145
Gln189
Glu166
Hydrogen bond from subgroups R1, R2, and R3
The remained hydrogen bond
11b
His164
Cys145
Gly143
Thr26
His163
Gln189
Glu166
Phe140
GC376
His163
Phe140
Cys145
His164
Gln189
Glu166
N3
Gly143
His163
Cys145
Phe140
Gln189
His164
Glu166
Thr190
11a
Cys145
Thr26
His164
His163
Glu166
Phe140
Boceprevir
Thr26
Asn142
Ser144
Gly143
His41
Cys145
His164
Glu166

Fig. S3

Fig. S3

Crystal structure of M^{pro} in complex with MG132. **a** Three-dimensional structure of SARS-CoV-2 M^{pro} in complex with MG132, in two different views. Cartoon representation of one protomer of the dimer is shown in pale cyan, and the other in wheat. Domains are labeled by Roman numerals. **b** Left panel, the *Fo-Fc* density map contoured at 1.6σ is shown around MG132 (blue mesh), Cys145 of M^{pro} (orange mesh), and the two waters (blue mesh). Right panel, the 2*Fo-Fc* electron density maps for residues involved in MG132 binding are shown as gray mesh and contoured at 1.6σ. **c** Molecular weights of M^{pro} and compound-treated M^{pro} determined by liquid chromatography−mass spectrometry (LC−MS). Ebselen is used as control. **d** The binding mode of MG132 to the active site of SARS-CoV-2 M^{pro} (upper panel) and yeast 20S beta5-D166N mutant (lower panel; PDB code: 5D0T). Proteins are shown as a gray surface. The side chains of the three Leu and the benzyl group of MG132 are in light blue, pale yellow, orange, and light pink, respectively. **e** Detailed view of the hydrogen bonds formed between SARS-CoV-2 M^{pro} and the indicated compounds. Hydrogen bonds were predicted by LigPlot+ v.2.2 (maximum distance set as 3.3 Å).

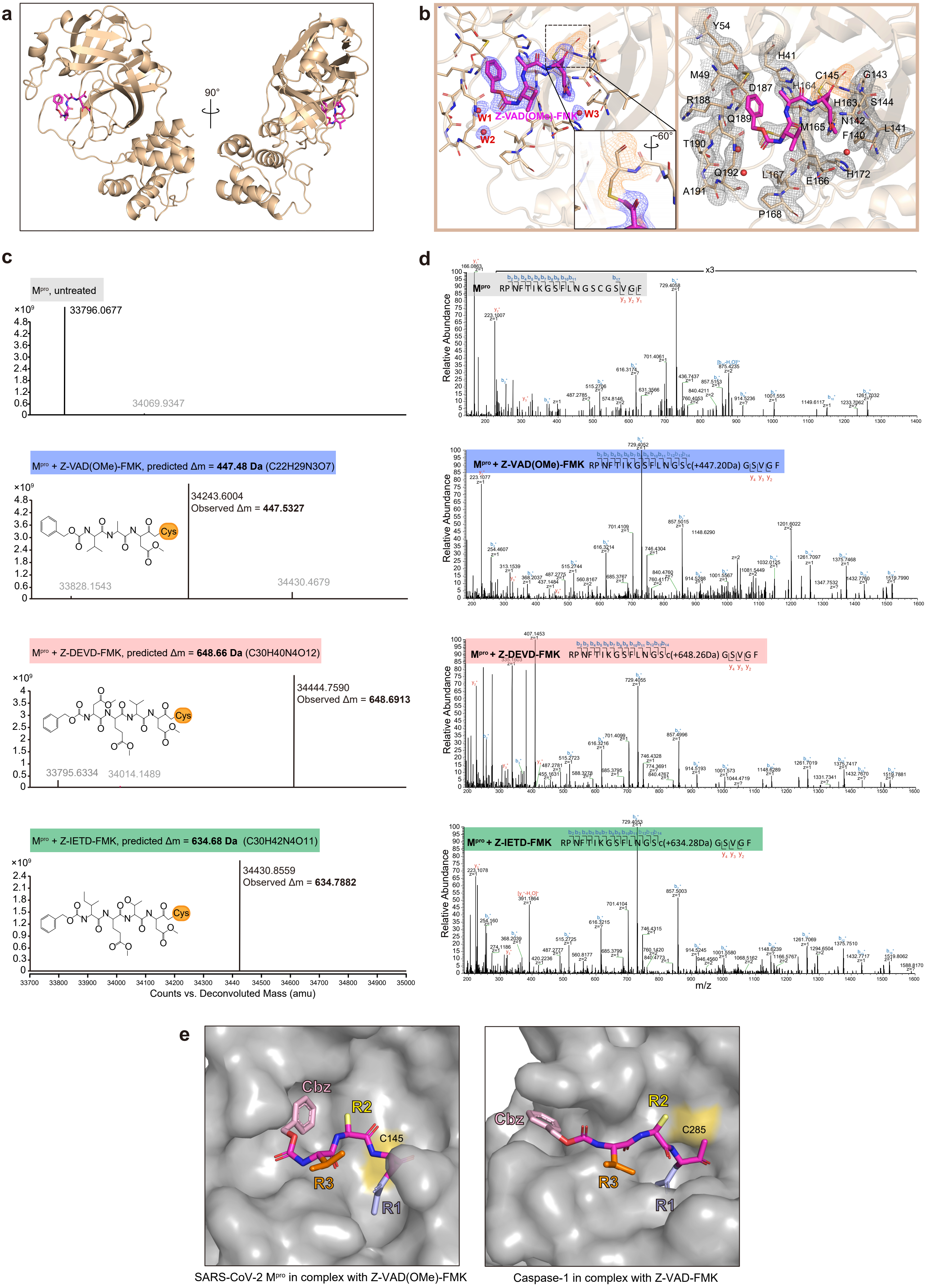
a
90°
b
W1
Z-VAD(OMe)-FMK
W3
W2
~60°
Y54
H41
M49
C145
G143
D187
H164
R188
H163
S144
Q189
N142
M165
F140
L141
T190
Q192
L167
H172
E166
A191
P168
c
Mpro, untreated
33796.0677
34069.9347
Mpro + Z-VAD(OMe)-FMK, predicted Δm = 447.48 Da (C22H29N3O7)
34243.6004
Observed Δm = 447.5327
33828.1543
34430.4679
Cys
Mpro + Z-DEVD-FMK, predicted Δm = 648.66 Da (C30H40N4O12)
34444.7590
Observed Δm = 648.6913
33795.6334
34014.1489
Mpro + Z-IETD-FMK, predicted Δm = 634.68 Da (C30H42N4O11)
34430.8559
Observed Δm = 634.7882
Counts vs. Deconvoluted Mass (amu)
d
x3
Mpro RPNFTIKGSFLNGSCGSVGF
Mpro + Z-VAD(OMe)-FMK RPNFTIKGSFLNGSc(+447.20Da) GSVGF
Mpro + Z-DEVD-FMK RPNFTIKGSFLNGSc(+648.26Da) GSVGF
Mpro + Z-IETD-FMK RPNFTIKGSFLNGSc(+634.28Da) GSVGF
Relative Abundance
m/z
e
Cbz
R2
C145
R3
R1
C285
SARS-CoV-2 Mpro in complex with Z-VAD(OMe)-FMK
Caspase-1 in complex with Z-VAD-FMK

Fig. S4

Fig. S4

Crystal structure of M^{pro} -Z-VAD(OMe)-FMK reveals a unique binding mode. **a** Three-dimensional structure of SARS-CoV-2 M^{pro} in complex with Z-VAD(OMe)-FMK, in two different view. M^{pro} and Z-VAD(OMe)-FMK are shown as wheat cartoon and magenta sticks, respectively. **b** Left panel, the *Fo-Fc* density map contoured at 1.6σ is shown around Z-VAD(OMe)-FMK (blue mesh), Cys145 of M^{pro} (orange mesh) and the three waters (blue mesh). Right panel, the 2*Fo-Fc* electron density map for residues involved in inhibitor binding is shown as gray mesh and contoured at 1.6σ. **c** Molecular weight of M^{pro} and Z-VAD(OMe)-FMK, Z-DEVD-FMK, and Z-IETD-FMK-treated M^{pro} determined by LC–MS. **d** Tandem MS/MS analysis of M^{pro} and compound-treated M^{pro}. A higher-energy collisional dissociation (HCD) MS/MS spectrum recorded on the $[M+H]^{3+}$ ion at m/z 696.68481 of the unmodified M^{pro} peptide RPNFTIKGSFLNGSCGSVGF, the $[M+H]^{3+}$ ion at m/z 912.77496 of the modified M^{pro} peptide RPNFTIKGSFLNGSCGSVGF harboring a modification(-C30H40N4O12) induced by Z-DEVD-FMK on Cys145, the $[M+H]^{3+}$ ion at m/z 908.11261 of the modified M^{pro} peptide RPNFTIKGSFLNGSCGSVGF harboring a modification(-C30H42N4O11) induced by Z-IETD-FMK on Cys145, and the $[M+H]^{3+}$ ion at m/z 845.75079 of the modified M^{pro} peptide RPNFTIKGSFLNGSCGSVGF harboring a modification(-C22H29N3O7) induced by Z-VAD(OMe)-FMK on Cys145. Predicted b- and y-type ions (not including all) are listed above and below the peptide sequence, respectively. **e** The binding mode of Z-VAD(OMe)-FMK to the active site of SARS-CoV-2 M^{pro} (left panel) and Z-VAD-FMK to caspase-1 (right panel; PDB code: 2HBQ). Proteins are shown in gray surface. The side chains of the three residues and benzyl group of Z-VAD(OMe)-FMK or Z-VAD-FMK are in light blue, pale yellow, orange, and light pink, respectively.

a

Compounds	IC_{50} (μM)	EC_{50} (μM)	Reference
**Z-VAD(OMe)-FMK**	0.59 ± 0.44	1.88 ± 0.52	The current study
**Z-DEVD-FMK**	2.80 ± 0.32	0.87 ± 0.10	The current study
**Z-IETD-FMK**	1.61 ± 0.05	0.64 ± 0.08	The current study
**N3**	Not available	16.77 ± 1.70	Jin et al., *Nature* (2020)
**11a**	0.05 ± 0.01	0.53 ± 0.01	Dai et al., *Science* (2020)
**11b**	0.04 ± 0.00	0.72 ± 0.09	Dai et al., *Science* (2020)
**Boceprevir**	4.13 ± 0.61	1.31 ± 0.58	Ma et al., *Cell Res.* (2020)
**GC-376**	0.03 ± 0.01	3.37 ± 1.68	Ma et al., *Cell Res.* (2020)
**Calpain inhibitor II**	0.97 ± 0.27	3.70 ± 0.69	Ma et al., *Cell Res.* (2020)
**Calpain inhibitor XII**	0.45 ± 0.06	0.78 ± 0.37	Ma et al., *Cell Res.* (2020)
**MI-09***	15.2 ± 0.4	0.86 ± 0.07	Qiao et al., *Science* (2021)
**MI-12***	19.0 ± 0.6	0.53 ± 0.07	Qiao et al., *Science* (2021)
**MI-14***	13.0 ± 0.3	0.66 ± 0.06	Qiao et al., *Science* (2021)
**MI-28***	9.2 ± 0.2	0.67 ± 0.06	Qiao et al., *Science* (2021)
**MI-30***	17.2 ± 0.6	0.83 ± 0.28	Qiao et al., *Science* (2021)
**MI-31***	30.0 ± 0.4	0.83 ± 0.28	Qiao et al., *Science* (2021)

** The 6 most potent M^{pro} inhibitors in Qiao et al. study were selected for presenting*

b

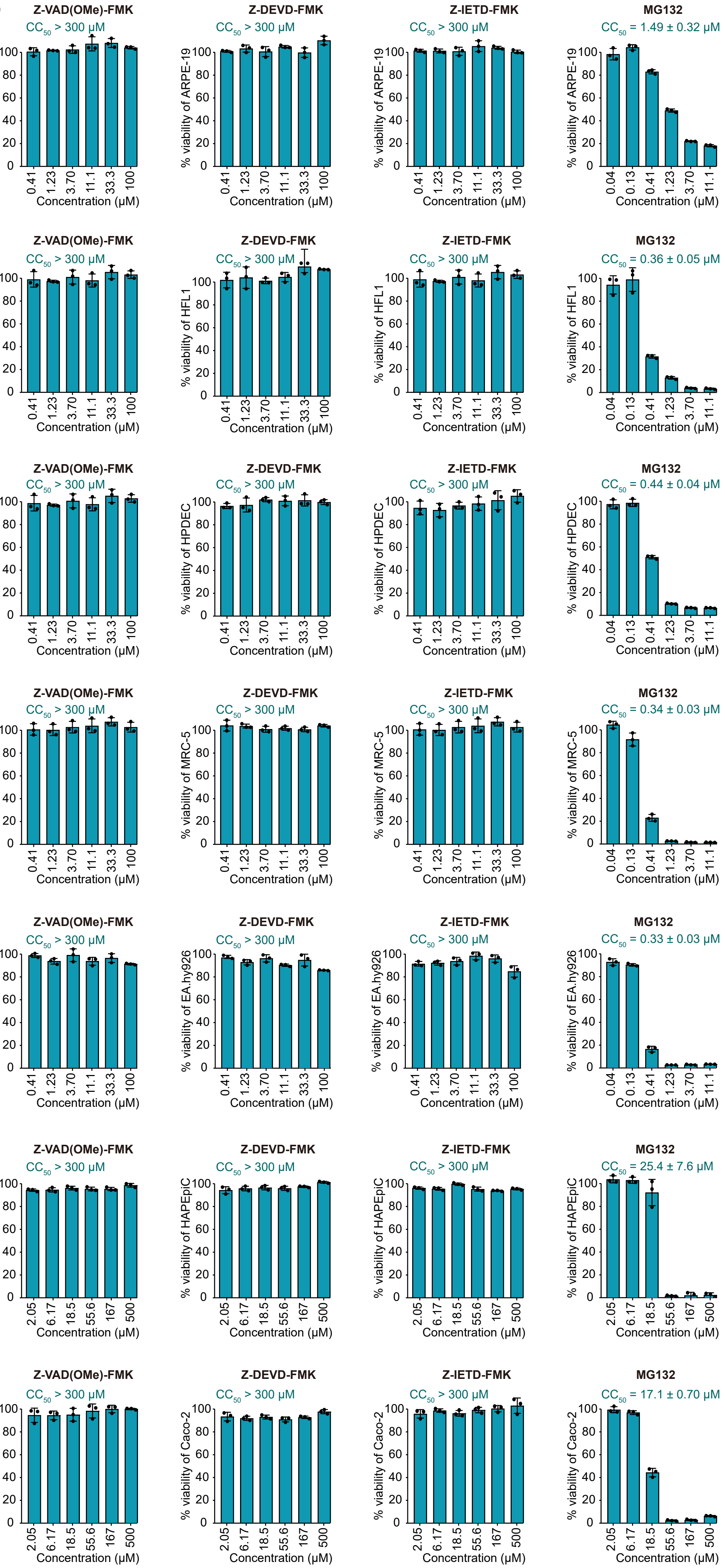

Fig. S5

Fig. S5

a List of IC_{50} and EC_{50} values of the SARS-CoV-2 M^{pro} inhibitors. The compiled EC_{50} values were all derived from antiviral experiments performed in Vero cells. **b** Cytotoxicity of the indicated compounds in the indicated cell lines determined by CCK8 assay. The Y-axe of the graphs represent mean % cell viability.

Caption for Tables S1 to S2

Table S1. M^{pro} apparent melting temperature upon treatment of the screened compounds (provided as Excel Files).

Table S2. Crystallographic data collection and refinement statistics (provided as Excel Files).
